# Current approaches to measuring high-intensity locomotor actions in adult male professional soccer. A scoping review

**DOI:** 10.5114/biolsport.2026.158302

**Published:** 2026-03-05

**Authors:** Paul McGrath, Damian Harper, Jill Alexander

**Affiliations:** 1 Leyton Orient Football Club; 2 Football Performance Hub, Institute of Coaching and Performance (ICaP), School of Health, Social Work and Sport, University of Lancashire, Preston, Lancashire, UK

**Keywords:** Acceleration, Deceleration, Global Positioning Systems, High-Speed Running, Maximum Demands, Sprinting

## Abstract

High-intensity locomotor actions (HILAs) including high-speed running (HSR), sprinting, accelerations, and decelerations are critical to performance and injury risk in professional soccer. Rapid technological and methodological developments since 2021 necessitate an updated synthesis of how these actions are quantified and interpreted in applied settings. A scoping review was conducted following PRISMA-ScR guidelines. Five databases (CINAHL, MEDLINE, SPORTDiscus, Academic Search Complete, Web of Science) were searched for English-language studies published between 22 March 2021 and 22 February 2025. Eligible studies included adult male professional soccer players and quantified HILAs in training and/or matches using Global Positioning Systems (GPS), Local Positioning Systems (LPS; sampling frequency > 10 Hz), or digital video-based tracking. Twenty studies met the inclusion criteria. Most used 10 Hz GPS units (n = 16) and applied absolute thresholds (n = 16) (e.g., HSR > 19.8 km · h^−^¹, sprinting > 25.2 km · h^−^¹, accelerations/decelerations > 3 m · s^−2^/ < −3 m · s^−2^), with only four adopting relative thresholds. Data filtering procedures were often underreported, and temporal normalisation was uncommon. Tactical (e.g., formation, playing style), temporal (e.g., match phase), and positional (e.g., role, field zone) contexts were rarely considered, and only one study integrated GPS with video analysis. Training typically under-replicated match demands, particularly for non-starters. Considerable methodological heterogeneity limited cross-study comparability. In conclusion, current monitoring practices for HILAs in professional soccer remain dominated by GPS and absolute thresholds, with limited individualisation and contextual integration. Future research should prioritise standardised threshold definitions, transparent data processing, and integration of video and GPS technologies to enhance ecological validity and applied impact.

## INTRODUCTION

High-intensity locomotor actions (HILAs) such as high-speed running (HSR), sprinting, accelerations, and decelerations, typically defined using absolute or relative velocity or acceleration thresholds, are central to performance in adult male professional soccer [1, 2]. These actions have become increasingly frequent and intense over recent decades [3–5], with clear links to match-defining moments [6, 7] and team outcomes [8]. Forecasts suggest that HSR distances (> 25.1 km · h^−^¹) in the English Premier League may increase by up to 40% between 2020 and 2030 [9], alongside rises in sprinting and high-intensity accelerative and decelerative efforts [8]. These trends reflect the increasing speed and tactical complexity of the modern game, which vary substantially by position. For example, wide players typically accumulate greater HSR, whereas forwards perform more repeated high-intensity accelerations and decelerations [5, 7, 10, 11]. Such positional heterogeneity challenges uniform threshold application and cross-study comparability, as fixed cut-offs may misrepresent the true intensity demands of specific roles.

There is a strong link between HILAs, injury risk, and performance load management [12–15], although evidence regarding sprintingrelated hamstring injury risk remains mixed. Some studies identify maximal sprinting as a key precipitating factor, whereas others emphasise the multifactorial nature of risk, highlighting eccentric strength, previous injury, and exposure history [16–19]. High-intensity decelerations generate ground reaction forces up to six times body weight, approximately twice those during maximal sprinting and elicit greater creatine kinase and soreness responses than steadystate running [18, 20, 21]. These eccentric demands have been associated with anterior cruciate ligament (ACL) injuries [22], and both over- and under-exposure may elevate injury risk [23]. Accelerations similarly impose substantial strain during transitional and pressing actions [24, 25] and are metabolically demanding [26]. Accordingly, accurate monitoring and profiling of HILAs across positions and contexts are essential for optimising training design, managing player load, and mitigating injury risk [27, 28].

A range of technologies are used to monitor HILAs. Global Positioning Systems (GPS) remain most common, offering portable and relatively low-cost solutions for quantifying external load and identifying peak exertion periods [29]. A sampling frequency of ≥ 10 Hz is recommended for reliable distance and speed measurement; however, accurately capturing accelerations and decelerations remains problematic. Rapid velocity changes may occur within time intervals shorter than the device’s sampling window, producing signal smoothing and underestimation of peaks. Moreover, acceleration and deceleration metrics are often derived from tri-axial accelerometers integrated within GPS units rather than solely from positional differentiation, which may introduce additional noise and limit sensitivity to brief, high-magnitude movements [30–34]. Local Positioning Systems (LPS) provide higher spatial and temporal resolution in environments where GPS accuracy may be compromised, such as stadiums or indoor facilities [35], and demonstrate superior validity for quantifying rapid speed changes during intense accelerations and decelerations [36, 37]. Video-based optical tracking systems (OTS), including Second Spectrum, TRACAB, and STATS SportVU, are widely used for tactical and physical analysis [38, 39]. While generally reliable for distance-based metrics, OTS often overestimate sprint demands compared with GPS [40, 41]; HSR and sprint distances can be 12–18% higher despite strong correlations (r^2^ > 0.99) [42]. However, no standardised agreement currently exists between GPS, LPS, and OTS technologies, each using distinct algorithms, filters, and smoothing parameters. Consequently, data cannot be used interchangeably across devices or competitions [43–45].

Threshold selection can be conceptualised along a continuum from fixed to fully individualised approaches, each with distinct implications for interpreting HILAs. Early studies primarily employed absolute thresholds (e.g., > 19.8 km · h^−^¹ for HSR, > 25.2 km · h^−^¹ for sprinting, > 3 m · s^−2^ for accelerations, < −3 m · s^−2^ for decelerations), which facilitated between-study standardisation but failed to account for inter-individual differences in physical capacity [21, 45–48]. Consequently, absolute thresholds may underestimate demands on faster players and overestimate those on slower players, obscuring meaningful workload differences across individuals and positions. Relative thresholds, defined as percentages of a player’s maximum sprint speed (MSS) or test-derived velocities (e.g., VIFT), improve individualisation and contextual accuracy but require regular testing and lack universal reference values, limiting comparability [24, 47, 48]. More recently, adaptive thresholding models that dynamically adjust based on rolling match or training data have emerged. These models aim to balance individual sensitivity with longitudinal consistency, offering a pragmatic bridge between absolute and relative approaches, though adoption remains limited [4, 14, 47, 49, 50].

Beyond threshold selection, temporal and contextual framing plays a crucial role in accurately capturing HILAs [49, 51]. Traditional analyses using whole-match averages underestimate physical intensity by including stoppages, substitutions, and other low-activity periods [52, 53]. In contrast, ball-in-play (BiP) analyses isolate active phases, providing a more accurate representation of true physiological and mechanical load and allowing clearer identification of peak and fluctuating demands. By excluding inactive time, BiP-derived values for HSR and sprinting are typically 10–20% higher than wholematch estimates [54–58], reflecting methodological superiority for quantifying genuine intensity. BiP analysis also facilitates integration with tactical phases (e.g., in-possession, out-of-possession, transitions), enhancing ecological validity and supporting more applied load prescriptions. Similarly, the choice of moving average window substantially influences how peak demands are quantified and interpreted. Shorter epochs (e.g., 30 s–1 min) are more sensitive to transient high-intensity bursts and provide greater ecological validity for capturing the most demanding passages of play [59, 60]. Longer epochs (e.g., 3–5 min) smooth fluctuations and may underestimate instantaneous load, though they better represent sustained work rates. Recent approaches combining rolling averages with cumulative time above key thresholds offer a more complete view of the volume–intensity relationship during match play [61, 62]. Despite their potential, few studies have contextualised HILAs through tactical phases or integrated GPS with video analysis to align physical outputs with match events [63], limiting ecological validity and applied utility.

Given the evolution of match demands, variability in measurement technologies, inconsistent thresholding practices, and limited contextualisation, there is a clear need to consolidate and critically evaluate current approaches to quantify HILAs in adult male professional soccer. A scoping review design was selected to comprehensively map this emerging evidence base, capturing methodological diversity and conceptual developments since the rapid expansion of wearable and optical technologies post-2021. Unlike systematic reviews, which assess intervention efficacy or effect size, the scoping approach enables identification of methodological inconsistencies, evidence gaps, and emerging trends across heterogeneous study designs. This review therefore aims to clarify how HILAs are operationalised, highlight underexplored areas such as adaptive thresholding and contextual integration, and provide a conceptual framework to guide future research and applied monitoring practices. Specifically, this scoping review seeks to (1) evaluate contemporary methodologies used to quantify HILAs in adult male professional soccer; (2) identify methodological inconsistencies, evidence gaps, and emerging trends; and (3) provide recommendations to support more standardised, individualised, and context-sensitive monitoring frameworks.

## MATERIALS AND METHODS

### Study Design

This scoping review was conducted in accordance with the Preferred Reporting Items for Scoping Reviews (PRISMA-ScR) guidelines [65]. The five-step methodological framework outlined by Arksey and O’Malley [66], and later refined by Levac et al. [67], was followed. These steps included: (1) identifying the research question, (2) identifying relevant studies, (3) study selection, (4) charting the data, and (5) collating, summarising, and reporting the results. This approach ensured methodological transparency and alignment with established scoping review protocols.

### Information Sources and Search Strategy

Five electronic databases (CINAHL, MEDLINE, SPORTDiscus, Academic Search Complete, and Web of Science) were systematically searched by the lead author (PM) to identify peer-reviewed articles published in English between 22 March 2021 and 22 February 2025. The search strategy was developed using the Population–Concept–Context (PCC) framework, in line with methodological recommendations for scoping reviews by Peters et al. [68]. Eligible studies included adult male professional soccer players (Population), focused on high-intensity locomotor actions (HILAs) (Context), and reported approaches to measuring these actions (Concept). This scoping review builds on the systematic review of worst-case scenarios by Rico-González et al. [64] and intentionally focused on research published after 22 March 2021 to capture recent methodological developments in tracking technologies and thresholding approaches. While this timeframe ensured the inclusion of contemporary evidence reflecting current practice, it may have excluded earlier foundational studies that provide methodological continuity across technological generations.

Search terms were refined through pilot searches, during which the titles, abstracts, and full texts of known relevant literature were screened to optimise sensitivity and specificity. This process led to the inclusion of additional synonyms for “high-intensity actions” (e.g., “locomotor” and “running demands”) and the refinement of Boolean combinations to ensure both thresholding and tracking terminology (e.g., “GPS,” “LPS,” “optical tracking”) were consistently captured across databases. Boolean operators “AND” and “OR” were used to construct the final search strategy (Table 1). The PCC framework also guided study inclusion and exclusion decisions (Table 2). The restriction to English-language publications was applied for feasibility; however, this introduces a potential language bias, which is acknowledged as a limitation.

**TABLE 1:** Search Strategy.

PCC	Key Search Terms	Related Search Terms
1. Population	Male Professional Soccer Players	“Professional football” **OR** “Professional soccer”

2. Context	High-Intensity Actions	“High speed*” **OR** “Sprint*” **OR** “Accel*” **OR** “Decel*”

3. Concept	Approaches to measuring high-intensity actions	“Monitoring” **OR** “Prescribing” **OR** “Workload” **OR** “WCS” **OR** “Worst Case Scenario” **OR** “Most Demanding” **OR** “Maximum” **OR** “Average” **OR** “Drill” **OR** “Training” **OR** “Game” **OR** “Match” **OR** “GPS” **OR** “Global Positioning System” **OR** “Video Technology” **OR** “Relative Speed” **OR** “Sampling frequency” **OR** “Data” **OR** “Tracking System” **OR** “Optical Tracking” **OR** “Metric” **OR** “Insight” **OR** “practice” **OR** “fixture” **OR** “artificial-intelligence” **OR** “AI”

Search Phrase	1 **AND** 2 **AND** 3	

**TABLE 2:** Study Inclusion-Exclusion Criteria.

	Inclusion Criteria	Exclusion Criteria
**1**	Original research articles, academic/peer-reviewed text and reviews	Magazines, surveys, opinion pieces, books, periodicals, editorials, conference abstracts, non-academic/non-peer reviewed text, grey literature

**2**	Data since 22/03/2021	Data obtained before 22/03/2021

**3**	Male soccer or soccer outfield professional players	Studies developed with players from other team sports, female players, youth players, goalkeepers and semi-professional players

**4**	Participants aged > 18 years of age	Participants aged < 18 years regardless if average age of participant population group is 18 years.

**5**	Used GPS / LPS systems (with sampling frequency > 10 Hz) or digital video based tracking	GPS units (with sampling frequency of < 10 Hz), any non-GPS or LPS system excluding digital video based tracking

**6**	Full text available in English	Cannot access full-text in English

**7**	“High-intensity actions” include high speed running (5.5 m/s- 7 m/s), sprinting (7 m/s+), high-intensity accelerations (> 3 m/s^2^), high-intensity decelerations (< 3 m/s^2^) aswell as relative speed bands (% of maximal capacity) used for the aforementioned metrics captuted during training and matches	Other actions described as “high-intensity actions” i.e. jumping

**GPS** = Global Positioning System, **LPS** = Local Positioning System

### Screening Strategy

All search results were imported into RefWorks and exported to Microsoft Excel (Microsoft, Redmond, WA, USA) for screening. Titles, abstracts, and methods were first screened by the lead author (PM) to assess eligibility. All potentially relevant studies were then subjected to full-text review. To minimise bias, two co-authors (DH and JA) independently reviewed the final pool of included studies. Any disagreements were resolved by discussion and consensus, with a third reviewer available if needed, although this was not ultimately required. While initial screening was conducted by a single reviewer for feasibility reasons, subsequent dual independent screening of full texts ensured alignment with PRISMA-ScR recommendations and reduced the potential for selection bias. Inter-rater reliability was maintained through regular cross-checking and consensus meetings between reviewers rather than formal kappa statistics, which is consistent with methodological guidance for scoping reviews. A total of 20 studies met the inclusion criteria and were retained for data extraction.

### Data Extraction

Data were extracted systematically using a structured Excel form based on key study characteristics, including participant demographics, competition level, and measurement devices. Extraction variables were selected in advance to address the review objectives and included: (1) study design and sample characteristics (age, playing standard, competitive level); (2) tracking technology specifications (device type, sampling frequency, manufacturer, validation evidence); (3) operational definitions and thresholds for HILA metrics (absolute, relative, or adaptive); (4) data processing procedures (filtering, smoothing, temporal normalisation); (5) contextual dimensions (tactical phase, positional role, match status, environment); and (6) integration of internal load indicators (heart rate, RPE, biochemical markers). These categories were chosen to allow systematic comparison of methodological practices and to identify consistencies, gaps, and emerging innovations in the measurement of HILAs across professional soccer studies. Where applicable, the distinction between absolute and relative measurement approaches was recorded to examine methodological trends.

To ensure consistency, absolute and relative measures were coded using pre-defined operational criteria within the data extraction sheet. Studies were classified as absolute if thresholds were fixed and independent of player capacity, and as relative if metrics were scaled to individual or positional benchmarks (e.g., %MSS or VIFTderived values). Coding decisions were independently verified by two reviewers (PM and DH), with discrepancies resolved through discussion until consensus was achieved. This process ensured standardisation and reproducibility in the extraction and classification of methodological approaches.

## RESULTS

### Search Results

**FIG. 1:**
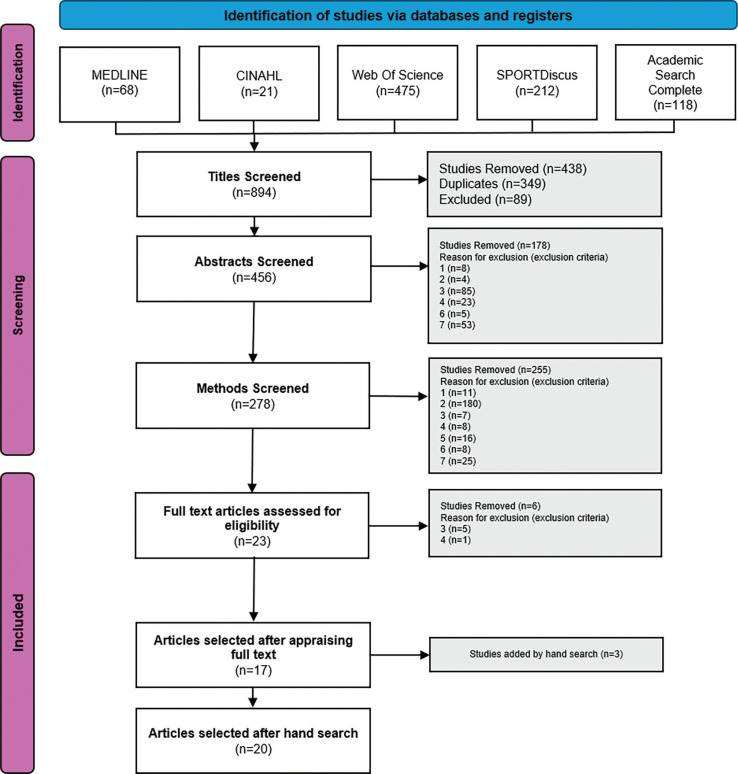
PRISMA flow diagram illustrating the identification, screening, eligibility, and inclusion process for study selection.

A total of 894 records were identified across the five databases, with 349 duplicates removed prior to screening. Following title, abstract, and methods screening, 23 full-text articles were assessed for eligibility. Six studies were excluded because participants were semiprofessional (n = 5) or under 18 years old (n = 1). An additional three studies were identified through hand-searching of reference lists. Consequently, 20 studies met all inclusion criteria and were retained for data extraction and synthesis (Figure 1).

### Study Characteristics

Table 3 summarises the characteristics of the included studies. All 20 studies used a cohort design and focused exclusively on adult male professional soccer players [63, 69–87]. All were published between 2022 and 2025, with 55% (n = 11) published in 2024 [63, 71, 72, 75, 77, 78, 80, 81, 84, 85, 87]. Sample sizes ranged from 10 to 3,211 participants, with most studies averaging 20–30 players aged 24–28 years.

**TABLE 3:** Summary of study characteristics and methodological details for included studies (n = 20).

Study	Study Design	Participant Characteristics (age; weight; height)	Competition Level (Country)	Measurement Details (GPS System, Digital video-based tracking)	Filtering (MED; Raw/Software)	Data Collected (Match / Training / Both)
Ammann et al. [69]	Cohort Study	25 males (age, weight, height NR)	1^st^ Division, Switzerland	10 Hz GPS (Apex Pro, STATSports; Sonra Software)	NR; Software	Match & Training

Padrón-Cabo et al. [70]	Cohort Study	10 males (26.52 ± 4.25 yrs; 73.47 ± 3.24 kg; 178.0 ± 6.36 cm)	Spain	10 Hz GPS (Playertek, Catapult; Vector Software)	NR; Software	Match & Training

Gonçalves et al. [63]	Cohort Study	24 males (26.3 ± 5.6 yrs; 75.3 ± 5.6 kg; 177.3 ± 5.6 cm)	4^th^ Division, Brazil	10 Hz GPS (Catapult S7; Vector Software); SBG Sports Software	NR; Software	Matches

Owen et al. [71]	Cohort Study	37 males (25 ± 4.1 yrs; 81.1 ± 6.7 kg; 181.9 ± 6.7 cm)	1^st^ Division, Scotland	10 Hz GPS (Catapult S7; Vector Software)	NR; Raw	Match & Training

Oliva-Lozano et al [72]	Cohort Study	26 males (27.3 ± 2.7 yrs; 78.8 ± 6.6 kg; 1.80 ± 0.1 m)	1^st^ Division, Hungary	10 Hz GPS (WIMU Pro, RealTrack Systems)	NR; Software	Matches

Aquino et al. [73]	Cohort Study	22 males (28.4 ± 4.9 yrs; 72.9 ± 7.1 kg; 1.78 ± 0.1 cm)	1^st^ Division, Brazil	10 Hz GPS (Polar Electro)	NR; Software	Matches

Beato et al. [74]	Cohort Study	24 males (27 ± 9 yrs; 79 ± 15 kg)	2^nd^ Division, United Sonra Kingdom Software)	10 Hz GPS (Apex Pro, STATSports; Sonra Software)	NR; Raw	Training

Falces-Prieto et al. [75]	Cohort Study	17 males (26.6 ± 4.2 yrs; 182.5 ± 6 cm; 75.29 ± 7.16 kg)	2^nd^ Division, Belgium	10 Hz GPS (WIMU PRO, RealTrack Systems)	NR; Software	Match & Training

Long et al. [76]	Cohort Study	25 males (26.3 ± 3.8 yrs; 76.9 ± 9.9 kg)	United Soccer League, America	10 Hz GPS (Apex Pro, STATSports; Sonra Software)	HSR > 1 s; Acc/ Dec > 0.5 s; Raw	Matches

Janusiak et al. [77]	Cohort Study	21 males (24.9 ± 3.2 yrs; 179.6 ± 5.5 cm; 76.1 ± 5 kg)	1^st^ Division, Poland	10 Hz GPS (Catapult S7; Vector Software)	NR; Software	Matches

Beato et al. [78]	Cohort Study	25 males (27 ± 9 yrs; 78 ± 14 kg)	EFL League 1, United Kingdom	10 Hz GPS (Apex Pro, STATSports; Sonra Software)	NR; Raw	Matches

Silva et al. [79]	Cohort Study	19 males (27.5 ± 4.6 yrs; 182 ± 6 cm; 73.5 ± 6.3 kg)	NR	15 Hz GPS (GPSports System, Australia)	NR; Software	Match & Training

Ponce-Bordón et al. [80]	Cohort Study	20 males (26.2 ± 5.3 yrs)	3^rd^ Division, Spain	18 Hz GPS (Apex Pod v 4.03, STATSports; Sonra Software)	HSR & Sprint > 1 s; Software	Training

Silva et al. [81]	Cohort Study	20 males (24.9 ± 4.0 yrs; 182.1 ± 7.5 cm; 75.1 ± 8.3 kg)	1^st^ Division, Portugal	10 Hz GPS (Catapult S7; Vector Software)	Sprint > 0.1 s; Raw	Matches

Beato et al. [82]	Cohort Study	25 males (27 ± 9 yrs; 78 ± 14 kg)	EFL League 1, United Kingdom	10 Hz GPS (Apex Pro, STATSports; Sonra Software)	NR; Raw	Training

Izzo et al. [83]	Cohort Study	25 males (24.1 ± 1.4 yrs; 79.8 ± 1.7 kg; 182.9 ± 0.6 cm)	3^rd^ Division, Italy	50 Hz GPS (K-Sport Universal STATS, Italy)	NR; Software	Match & Training

Bortnik et al. [84]	Cohort Study	31 males	1^st^ Division, Israel	10 Hz GPS (Catapult X7; Vector Software)	NR; Software	Match & Training

Asian-Clemente et al. [85]	Cohort Study	25 males (21.9 ± 1.9 yrs; 177.9 ± 5.2 cm; 75.5 ± 4.8 kg)	1^st^ Division, Spain	10 Hz GPS (WIMU Pro, RealTrack Systems)	NR; Software	Training

Castellano et al. [86]	Cohort Study	3,211 players CD: 592; FB: 627; CM: 822; WN: 515; FW: 655)	1^st^ Division (Germany, France, Spain, United Kingdom, Italy)	Digital Video Tracking (SkillCorner^®^)	NR; Raw	Matches

Silva et al. [87]	Cohort Study	42 males (26.7 ± 4.2 yrs; 74.5 ± 6.0 kg; 181.7 ± 6.3 cm)	1^st^ Division, Portugal	10 Hz GPS (Catapult S7; Vector Software)	NR; Raw	Training

GPS = Global Positioning System, MED = Minimal Effort Duration, NR = Not Reported, CD = Central Defender, FB = Full Back, CM = Central Midfielder, WN = Winger, FW = Forward

**TABLE 4:** Threshold definitions and key findings of included studies (n = 20).

Study	High Speed Running (Absolute or Relative)	Sprinting (Absolute or Relative)	Accelerations (Absolute or Relative)	Decelerations (Absolute or Relative)	Key Findings
Ammann et al. [69]	Relative (> 55% V_Max_)	Relative (> 70% of V_Max_)	Absolute (> 4 m/s^2^)	(Absolute < -4 m/s^2^)	Combined internal and external monitoring better reflected player load. HIIT drills increased HSR and time ≥ 90% HRMax more than SSGs. VMax calculation method not reported.

Padrón-Cabo et al. [70]	Absolute (19.8–25.1 km/h) & Relative (86.99–110% VIFT & 55–74.99% MSS)	Absolute (> 25.2 km/h) & Relative (> 110% VIFT & > 75% MSS)	NR	NR	Speed thresholds were individualised using 30–15 IFT and MSS. No clear performance difference between relative and absolute thresholds.

Gonçalves et al. [63]	Absolute (19.8–25.1 km/h)	Absolute (≥ 25.2 km/h)	Absolute (> 2 m/s^2^; meters/minute)	Absolute (< -2 m/s^2^; meters/minute)	Match context (IP, OOP, T2A, T2D) significantly influenced HILAs.

Owen et al. [71]	Absolute (> 5.5 m/s)	NR	Absolute (> 3 m/s^2^)	NR	HSR, HI accelerations, and %VMax were lowest on MD-1. Tracking within-player variability helped assess training load fluctuations.

Oliva-Lozano et al [72]	NR	Absolute (> 25.2 km/h)	Absolute (> 3 m/s^2^)	Absolute (< -3 m/s^2^)	Playing position influenced all metrics. Most HILAs occurred in the first 15 minutes.

Aquino et al. [73]	Absolute (19.8–25.1 km/h)	Absolute (> 25.2 km/h)	Absolute (≥ 3 m/s^2^)	Absolute (≤ -3 m/s^2^)	More HSR occurred during draws than wins. Weaker opponents triggered higher sprint demands.

Beato et al. [74]	Absolute (> 5.5 m/s)	Absolute (> 7 m/s)	Absolute (> 3 m/s^2^)	Absolute (< -3 m/s^2^)	LSGs best replicated match-like HSR and SD, though actual match intensity remained higher.

Falces-Prieto et al. [75]	Absolute (21–24 km/h)	Absolute (> 24 km/h)	Absolute (> 4 m/s^2^)	Absolute (< -4 m/s^2^)	HSR and intensity increased from weeks 1–3 in pre-season, then dropped in week 6. Most Accelerations and Decelerations occurred in weeks 2–3.

Long et al. [76]	Absolute (> 5.5 m/s)	Absolute (> 7 m/s)	Absolute (> 3 m/s^2^)	Absolute (< -3 m/s^2^)	STs and FBs recorded more HSR/SD than CDs and CMs. STs covered more SD in wins. Defenders had higher HSR in draws.

Janusiak et al. [77]	Absolute (19.81–25.2 km/h)	Absolute (> 25.2 km/h)	NR	NR	Substitute midfielders showed higher HSR than starters. Substitute forwards had more SD than midfielders.

Beato et al. [78]	Absolute (> 5.5 m/s)	Absolute (> 7 m/s)	Absolute (> 3 m/s^2^)	Absolute (< -3 m/s^2^)	Player position affected physical output, while match result and location did not.

Silva et al. [79]	NR	NR	Absolute (> 4 m/s^2^)	Absolute (< -4 m/s^2^)	Fullbacks executed x4 more HI decelerations in training than in matches.

Ponce-Bordón et al. [80]	Absolute (> 21 km/h)	Absolute (> 24 km/h)	Absolute (> 3 m/s^2^)	Absolute (< -3 m/s^2^)	Artificial turf resulted in higher HSR and SD than natural grass.

Silva et al. [81]	NR	Relative (> 80% of V_Max_)	Relative (> 75% of Max Acceleration)	Relative (> 75% of Max Deceleration)	More ball touches and accurate passes reduced SD, whereas dribbles increased it. Max values were derived from inseason match data.

Beato et al. [82]	Absolute (> 19.8 km/h)	Absolute (> 25.2 km/h)	Absolute (> 3 m/s^2^)	Absolute (< -3 m/s^2^)	MSGs led to more high-intensity actions than LSGs or SSGs. Player position affected HSR and HI decelerations.

Izzo et al. [83]	Absolute (> 20 km/h)	Absolute (> 25 km/h)	Absolute (> 3 m/s^2^)	NR	A Training/Match ratio of 1.7–2.1 for SD is optimal for adaptation and injury prevention.

Bortnik et al. [84]	Absolute (> 19.8 km/h)	Absolute (> 25.2 km/h)	Absolute (> 3 m/s^2^)	Absolute (< -3 m/s^2^)	Max accelerations and decelerations were not replicated in training. Non-starters lacked max intensity exposure in matches.

Asian-Clemente et al. [85]	Absolute (> 21 km/h)	NR	Absolute (> 3 m/s^2^)	Absolute (< -3 m/s^2^)	Positional games required more HSR, accelerations, and decelerations than possession-based drills.

Castellano et al. [86]	Absolute (> 20 km/h)	Absolute (> 25 km/h)	Absolute (> 3 m/s^2^)	Absolute (< -3 m/s^2^)	Tactical and physical profiles varied by country, reflecting different positional demands.

Silva et al. [87]	NR	NR	Relative - High (> 75%) Moderate (25–50%) Low (25–50%) Very low (< 25%)	Relative - High (> 75%) Moderate (25–50%) Low (25–50%) Very low (< 25%)	Relative thresholds were derived Relative - High from peak efforts in training and (> 75%) matches across four micro-cycles.

IFT = Intermittent Fitness Test, MSS = Maximum Sprinting Speed, HI = High-Intensity, HILAs = High-Intensity Locomotor Actions, HSR = High-Speed Running, SD = Sprint Distance, IP = In Possession, OOP = Out Of Possession, T2A = Transition To Attack, T2D = Transition To Defend, LSG = Large-Sided Game, MSG = Medium-Sided Game, SSG = Small-Sided Game, CD = Central Defenders, FB = Full Backs, CM = Central Midfielders, ST = Strikers

Geographically, studies included players competing in Spain [70, 80, 85, 86], the United Kingdom [74, 78, 82, 86], Brazil [63, 72], Switzerland [69], Scotland [71], Hungary [72], Belgium [75], the United States [76], Poland [77], Portugal [81, 87], Italy [83, 86], Israel [84], Germany, and France [86]. Studies were predominantly from top-tier [69, 71–73, 77, 81, 84, 86, 87] adult male professional leagues, with only one study [79] not reporting what geographical location of the participants. Five studies examined training data only [74, 80, 82, 85, 87], eight focused exclusively on match data [63, 72, 73, 76, 77, 78, 81, 86], and seven included both training and match contexts [69, 70, 71, 75, 79, 83, 84].

### Tracking Technologies and Data Collection

GPS technology was used in all studies except one [86], which employed video-based tracking. Sixteen studies used 10 Hz GPS [62, 69–78, 81, 82, 84, 85, 87], one used 15 Hz [79], one 18 Hz [80], and one 50 Hz [83]. Data filtering methods were explicitly reported in two studies (10%) [76, 80], while the remaining 18 (90%) did not specify their filtering or smoothing procedures, limiting reproducibility and cross-study comparability.

Most studies (n = 16) used absolute thresholds to define HILAs [63, 71–86], typically adopting values of > 19.8 km · h^−^¹ for high-speed running, > 25.2 km · h^−^¹ for sprinting, and > 3 m · s^−2^ and < –3 m · s^−2^ for accelerations and decelerations, respectively. Two studies applied relative thresholds only [81, 87], with one defining high-speed running and sprinting based on individual maximum sprinting speed [81], and the other defining acceleration and deceleration based on the maximum values achieved across a micro-cycle [87]. Two studies [68, 70] employed a combination of absolute and relative thresholds. The limited adoption of relative thresholds (n = 4; 20%) highlights an ongoing methodological gap between recommended and applied practices. Although relative and adaptive approaches offer greater individualisation by accounting for interplayer variability, their implementation remains uncommon, likely due to the additional testing burden and lack of consensus on standardised cut-points. This methodological disparity restricts comparability across studies and limits the translational value of research for applied load monitoring.

In terms of reporting, most studies (n = 17) presented cumulative values across entire matches or sessions [63, 70–73, 75–81, 83, 85–87], whereas only three normalised data to per-minute metrics [74, 82, 84]. The limited adoption of time-normalised reporting complicates cross-study comparisons, particularly between competitions with different match durations, stoppage times, or data segmentation methods. Without standardised temporal scaling, workload metrics (e.g., total HSR or sprint distance) can appear inflated or underestimated depending on match length, reducing the interpretability and external validity of findings. Establishing consistent time-normalised reporting practices would therefore enhance comparability and practical translation of results across leagues and contexts.

Considerable heterogeneity was observed in how studies defined and operationalised high-intensity locomotor actions. Thresholds for HSR ranged from > 18.0 to > 25.2 km · h^−^¹, sprinting from > 23.0 to > 30.6 km · h^−^¹, and accelerations from > 2.5 to > 3.5 m · s^−2^. Deceleration thresholds showed similar variation, ranging from < –2.5 to < –4.0 m · s^−2^. Only four studies applied relative or adaptive methods to define these categories [69, 70, 81, 87].

This definitional variability represents a major evidence gap, as inconsistent thresholding and data processing practices preclude meta-analytic comparison and limit the formulation of generalisable benchmarks. The lack of consensus-based standardisation across studies highlights the need for future methodological frameworks or Delphi processes to harmonise HILA definitions and enable more structured evidence mapping within this domain.

## DISCUSSION

The aim of this study was to identify and synthesise prevailing methods for quantifying HILAs in adult male professional soccer. Findings revealed widespread reliance on GPS technologies, limited use of relative or adaptive thresholds, and inconsistent data-processing procedures. While GPS remains the predominant technology, variability between device manufacturers, firmware versions, and proprietary filtering algorithms further complicates cross-study comparisons. Even when sampling frequencies are identical, data output can differ substantially depending on the manufacturer’s smoothing, interpolation, and satellite correction procedures, leading to systematic bias, particularly for short-duration, high-acceleration movements where latency or signal noise is more pronounced. Standardising validation protocols and promoting manufacturer transparency regarding data-processing algorithms would improve reliability and facilitate more meaningful comparisons across studies and contexts.

Studies using relative thresholds demonstrated greater sensitivity to individual differences by scaling metrics to player capacity (e.g., maximal sprint speed, VIFT, or peak match-play outputs) [69, 70, 81, 87]. These approaches enhanced contextual relevance in load monitoring [48, 63], yet only four studies implemented them, and none consistently across all HILAs. Most research still applied absolute cut-offs, overlooking inter-individual variability and underestimating player-specific demands. Although widely advocated, individualisation remains infrequently applied, revealing a clear gap between methodological recommendations and practice.

Contextual and tactical factors such as match status, opposition strength, and tactical phase substantially influence the occurrence of HILAs [63, 81, 87]. However, few investigations incorporated these variables when quantifying match demands [73, 76, 78, 80]. Of the 20 studies included, only one [63] explicitly integrated GPS data with video-coded tactical information, while three others [73, 76, 78] reported partial contextual descriptors (e.g., opposition level or match outcome) without formal analytical integration. The remaining studies did not include contextual variables, confirming that contextual integration remains the exception rather than the norm. Environmental and situational conditions also modulate external load, with greater sprint volumes observed on artificial turf and higher high-speed running against weaker opponents [73, 80]. Positional and tactical demands further shape activity profiles; wingers and full-backs typically accumulate greater distances during inpossession phases, whereas defenders perform more recovery sprints when out of possession [55, 88].

The limited use of integrated GPS–video systems likely reflects several practical and logistical barriers. Synchronising positional and tactical datasets requires advanced software infrastructure and technical expertise, while access to high-fidelity optical data is often restricted by commercial rights holders. Data integration also increases analytical complexity and time requirements, particularly when aligning event and tracking timelines. Overcoming these barriers will require collaborative partnerships between researchers, technology providers, and professional clubs, alongside open-source frameworks to streamline multi-source integration. To facilitate systematic inclusion of positional and tactical-phase variations, future research should employ multi-level monitoring frameworks that align physical metrics with contextual game states. For example, combining GPS and video-derived data enables event-based segmentation (e.g., in-possession, out-of-possession, transitions) and position-specific profiling. Standardising positional groupings (e.g., central defenders, wide players, forwards) and linking these to phase-specific demands would enhance comparability and advance applied load modelling. Recent benchmark analyses by Bradley [89] further contextualise HILA profiles across major international tournaments, providing reference points for interpreting current findings.

A consistent discrepancy between training and match demands was evident, particularly for sprinting and high-intensity accelerations. Four studies [74, 75, 79, 82] reported that training underreplicated match intensities, with non-starters experiencing reduced exposure to high-intensity workloads [63]. Many drills also failed to reproduce peak match demands [79, 85], although some evidence suggested that training-to-match ratios for specific HILA metrics (e.g., sprint distance) may optimise adaptation [83]. Insufficient exposure to match-level intensities can impair readiness and elevate soft-tissue injury risk, highlighting the importance of targeted training and compensatory conditioning. Incorporating contextual elements such as tactical phase and positional role could improve training specificity and ecological validity.

Positional and temporal variations in HILA output were also evident. Strikers and full-backs generally recorded higher high-speed running and sprint distances than central defenders and midfielders [76], while greater frequencies of HILAs occurred during the early phases of matches [72]. These patterns indicate that positional role and match period influence physical intensity, with implications for load management, substitution timing, and recovery planning. Training interventions could replicate the most demanding positional and temporal scenarios observed in match play – for example, exposing wide players to repeated sprint sequences reflecting in-possession transitions, and central defenders to short, high-force decelerations during out-of-possession phases. Embedding such contextual conditioning within weekly micro-cycles and small-sided games would align tactical intent with physical stimulus and optimise performance transfer.

Marked methodological heterogeneity persisted across studies. Threshold definitions, filtering procedures, and reporting standards varied considerably, with key methodological details often omitted. This variability echoes longstanding calls within sports science for greater methodological consensus. Comparable standardisation initiatives, such as the GPS consensus statement and injury surveillance frameworks, have shown how shared definitions and reporting checklists improve reproducibility. A Delphi-based consensus process involving researchers, practitioners, and technology providers could harmonise HILA thresholds, filtering practices, and contextual reporting to strengthen both transparency and applied translation. At present, optical tracking systems remain unvalidated by FIFA for measuring accelerations and decelerations, although ongoing improvements in sampling frequency and algorithm precision may help close this gap. It is also important to note that acceleration and deceleration metrics are derived from tri-axial accelerometers integrated within GPS devices rather than from the GPS signal itself, ensuring more accurate capture of high-frequency movement changes [45].

Only four investigations [69, 70, 81, 87] applied relative thresholds, and just two extended these to accelerations and decelerations [81, 87], limiting comparability and interpretability. Given their strong association with mechanical load and neuromuscular stress, accelerations and decelerations represent critical indicators of external load that warrant greater emphasis in future research. A major limitation concerns the limited integration of internal load measures with external data. Despite growing recognition that “intensity” cannot be fully captured through mechanical metrics alone [90], only one study included HR data ≥ 90% HR_max_ [69]. This overreliance on external metrics provides an incomplete representation of physiological strain. Furthermore, contextual, perceptual, and tactical dimensions were inconsistently reported, and longitudinal designs were scarce, restricting understanding of internal–external load relationships and adaptive responses to repeated high-intensity exposure. Finally, the restriction to English-language publications may have introduced language bias, potentially excluding relevant non-English studies.

## CONCLUSIONS

This scoping review highlights the central role of HILAs in adult male professional soccer while exposing substantial methodological fragmentation. Although GPS remains the dominant monitoring tool, absolute speed thresholds are the most commonly applied across studies, yet inconsistent threshold definitions, filtering procedures, and reporting standards continue to limit cross-study comparability and applied impact. A critical gap concerns the limited inclusion of internal load metrics. Reliance on external outputs alone neglects the physiological strain associated with HILAs and weakens understanding of player adaptation and fatigue. Integrating heart rate, perceptual, and biochemical measures alongside external data is essential to capture the full spectrum of player demands.

From an applied perspective, this integration could be achieved by synchronising internal and external metrics within unified monitoring platforms. For instance, combining GPS-derived external loads with heart rate or session-RPE data in the same time domain would enable practitioners to assess physiological efficiency (internal-to-external load ratios) and detect early signs of maladaptation. Incorporating biochemical or hormonal markers, where feasible, could further contextualise recovery and fatigue status. Developing standardised protocols for multi-modal data capture and aligning collection windows across technologies would improve consistency across clubs and research settings. However, feasibility and cost remain key considerations, particularly regarding GPS–video integration, which, despite its analytical value, may be limited by resource availability and technical expertise.

Future research should prioritise multi-modal, context-aware monitoring frameworks that combine internal, external, and tactical dimensions. To address practical constraints in elite soccer, hybrid research–practice collaborations embedded within existing club workflows are encouraged. Longitudinal observational designs across competitive seasons would enable repeated internal–external load integration without disrupting team operations. Cross-club data-sharing initiatives or anonymised central databases could enhance sample diversity and ecological validity, while lightweight, automated data pipelines linking GPS, RPE, and tactical video data may reduce practitioner workload and improve feasibility.

Recent technological and organisational advances are enhancing the practicality of such approaches. Commercial platforms such as Catapult OpenField [31], STATSports Sonra [32], and Kinexon PERFORM [33] now enable simultaneous synchronisation of GPS, inertial, and physiological data. Moreover, initiatives such as UEFA’s Football Research Programme [91] and FIFA’s EPTS Working Group [92] are developing standardised data protocols and validation frameworks that will facilitate harmonised, context-rich monitoring systems in professionalsoccer.

A progressive methodological roadmap should begin with the standardisation of threshold definitions, followed by systematic contextual integration and large-scale validation of hybrid tracking systems. Such integration, supported by transparent data reporting, is vital for advancing interpretation, training precision, and performance optimisation. Among these priorities, establishing consistent threshold definitions represents the most immediate and critical step, without a shared quantification basis, subsequent contextual or multimodal advances risk inconsistency and limited comparability. Once methodological alignment is achieved, contextual and tactical integration can meaningfully enhance ecological validity and applied translation.

## Data Availability

The data that support the findings of this study are available from the corresponding author upon request.
